# Barriers and facilitators to the implementation of an early intervention in psychosis service in three demonstration sites in Ireland

**DOI:** 10.1186/s12913-023-09585-3

**Published:** 2023-06-19

**Authors:** C.D. Darker, G. Nicolson, H. Reddon, K. O’Connor, R. Jennings, N. O’Connell

**Affiliations:** 1 Discipline of Public Health and Primary Care, Institute of Population Health, School of Medicine, Trinity College Dublin, Dublin, Ireland; 2 Health Promotion and Improvement Department, HSE Health and Wellbeing, 1st Floor Old National Ambulance Training Building, St Marys Hospital Campus, Phoenix Park, Dublin 20, Chapelizod, D20 TY72 Ireland; 3 Department of Medicine, University of British Columbia, Vancouver, Canada; 4 National Clinical Programme for Early Intervention in Psychosis, Health Service Executive Dublin, Dublin, Ireland; 5 Rise, South Lee Mental Health Services, Cork & Department of Psychiatry, University College Cork, Cork, Ireland; 6 Sexual Health and Crisis Pregnancy Programme, HSE Health and Wellbeing, Strategy and Research, 89-94 Capel St, Dublin 1, Dublin, D01 P281 Ireland

**Keywords:** Psychosis, Early intervention, Qualitative, Implementation, Process evaluation, MRC framework

## Abstract

**Background:**

Programmes for early intervention (EIP) in psychosis for people experiencing a first episode of psychosis (FEP) have been found to be both clinically and cost effective. Following the publication of a new EIP model of care (MoC) in Ireland, the aim of this research is to describe how people participated in and responded to the MoC including service users, family members, HSE clinical staff and HSE management.

**Methods:**

Qualitative design using the UK Medical Research Council’s process evaluation framework. Purposive sampling techniques were used. A total of N = 40 key informant semi-structured interviews were completed which included clinical staff (N = 22), health service managers and administrators (N = 9), service users (N = 8) and a family member (N = 1). Thematic analyses were conducted.

**Results:**

Unique features of the EIP service (e.g., speed of referral/assessment, multidisciplinary approach, a range of evidence-based interventions and assertive MDT follow up) and enthusiasm for EIP were identified as two key factors that facilitated implementation. In contrast, obstacles to staff recruitment and budget challenges emerged as two primary barriers to implementation.

**Conclusions:**

The findings from this research provide real world insights into the complexity of implementing an innovative service within an existing health system. Clear and committed financial and human resource processes which allow new innovations to thrive and be protected during their initiation and early implementation phase are paramount. These elements should be considered in the planning and implementation of EIP services both nationally in Ireland and internationally.

**Supplementary Information:**

The online version contains supplementary material available at 10.1186/s12913-023-09585-3.

## Background

Psychotic disorders and in particular schizophrenia, typically emerge during sensitive developmental stages such as adolescence and young adulthood. Schizophrenia can be chronic and lead to lifelong disability [1]. The estimated financial burden of schizophrenia in terms of direct costs of treatment and care, and indirect costs of informal care is significant [2]. There has been evidence that a delay in receiving appropriate treatment decreases the likelihood or the degree of recovery [3]. First episode psychosis (FEP) can adversely affect, and is associated with a pronounced decline in education, employment, and social functioning [4]. Early intervention in psychosis (EIP) teams aim to improve the short and longer term outcomes by targeting the early onset of psychosis by providing rapid referral and assessment pathways, providing both pharmacological and psychosocial treatments, involving families in care planning and protecting social support networks [5]. EIP is associated with improved clinical and functional outcomes at 5 years follow up, including depressive and cognitive measures as well as global and social functioning [6]. It is noted that these improved outcomes for EIP compared with standard care within community mental health teams (CMHTs) is attributed to EIP services having lower caseloads, which facilitate more intensive engagement with service users and their families, and can ensure better access to psychological and social support team members to allow EIP teams to offer a range of treatment options for service users [7]. Systematic reviews of EIP economic analyses have found EIP services are cost-effective compared with standard care for FEP [8]. EIP services were implemented as early as the 1980s in Australia, in the early 2000s in the United Kingdom, and in 2004 in Canada. In Ireland, EIP was first introduced in 2005 in a single setting in Dublin [9], a second EIP service started in 2012 in a more rural setting from a modified community rehabilitation team [10]. Both services were funded by grants and research initiatives which restricted implementation of an EIP service nationally [11]. Such challenges can be traced back to the chronic underfunding of public mental health services in Ireland, which has been identified as a systematic barrier to implementation of EIP internationally [12]. EIP is one of five National Clinical Programmes (NCPs) in the Health Service Executive in Ireland. The overall implementation of the NCP’s were investigated previously to determine the enablers and barriers to implementation generally across programmes [13]. A Model of Care (MoC) for EIP services was published by the NCP in 2019 [14]. This new MoC described how EIP Care should be delivered in Ireland. It recommends two service delivery models- standalone EIP teams for large urban areas (> 200,000 population) or “Hub and Spoke” EIP teams in more rural and dispersed populations. As there was a recognition that the Hub and Spoke model of service delivery was likely to apply to the vast majority of the Irish demographic and as a service delivery model it has less evidence base the HSE funded three Hub and Spoke ‘Demonstration Sites’ in Cork, South Lee (Population 200,000- Mix of urban, suburban and rural), Meath (Population 160,000- Mix of urban and rural), Sligo/ Leitrim (Population 115,000- largely rural population). The overall evaluation utilised three methodologies, which included, desk based review of existing EIP documentation within the service; quantitative data from demonstration sites and qualitative interviews [15].

There is value in the monitoring and identification of common issues, challenges and facilitative factors in implementing new EIP services in real world clinical settings [16]. A survey of implementation of EIP services in Italy found wide variability in the distribution of EIP services, partial fidelity to guidelines relating to medication prescribing but strong provision of structured psychotherapy and psychoeducation relating to psychosis [17]. Evaluation of EIP in Switzerland found that a key facilitative factor included the development of high quality case management where team members have a clearly identified role and specific competencies to focus on FEP [18]. A recent qualitative study conducted in the US with clinicians providing care for patients with FEP found that there were provider level barriers (e.g., additional demands on time that this type of care model requires such as building relationships and rapport with patients) and organisational level barriers (e.g., creating and maintaining expanded referral pathways from the community into the service) when trying to implement this type of coordinated service. Our study expands upon this work by exploring both facilitative factors and barriers to implementing an EIP model of care (MoC) into an existing public health service (HSE; Health Service Executive) in Ireland [14]. The current study was qualitative in nature and aims to understand implementation processes, and how key stakeholders, such as clinical team members, health service managers, service users and family members, responded to the implementation of a new EIP MoC in three demonstration sites in Ireland. This will provide a description of intentional and unintentional differences in delivery, and provide an opportunity to look at the contextual factors that may mediate the relationship.

## Methods

### Design

A process evaluation design, based upon the UK Medical Research Clinical (MRC) guidelines, was employed to investigate the implementation of the new EIP MoC [19]. The implementation of a new MoC represents a complex intervention, especially when looking to integrate this into an established healthcare service and setting. This study provides a description of barriers and facilitative factors for implementation. Ethical approval was granted by the Royal College of Physicians of Ireland (reference: RCPI RECSAF 79). Qualitative interviews were conducted at three demonstration sites which were attempting to implement the new MoC. The interviews explored how stakeholders participated in, responded to and experienced the MoC. The interviews were specifically interested in this new MoC and care was taken to ensure that EIP was the focus of each interview, rather than more generic mental health improvements in other settings.

### Setting and EIP model of care programme description

Three demonstration sites were chosen from an open application call by the NCP to test the ‘Hub and Spoke’ model in practice. Each demonstration site served areas which included rural communities with geographically dispersed populations. In this context, a Hub and Spoke model for rural areas and towns with populations below 200,000 persons is considered appropriate. The role of the Hub is to provide leadership, supervision, and provision of complex FEP assessments and interventions, other functions include governance and quality assurance of EIP delivery. The Spokes are embedded within the CMHTs and receive support and guidance from the ‘Hub’. A recent critical review found evidence that clinical outcomes of rural EIP teams demonstrate positive outcomes of a Hub and Spoke model on reducing hospital admissions, psychotic symptoms and improving quality of life [20]. The EIP MoC defines the staffing and the skill mix of the EIP teams- the funding allocated to the teams to recruit staff was aligned with this MOC. The posts in the EIP teams are non-discipline specific but rather they are skill specific e.g. Cognitive Behavioural Therapy for psychosis (CBTp) clinician, behavoiural and family therapist (BFT) clinician and keyworker. These posts could be filled by any mental health professional who has completed the required training and demonstrated the required competence. For example there were nurses, occupational therapists, social workers and psychologists all employed as CBTp clinicians across the EIP teams. There was a peer support worker in one of the EIP teams.

### Participants

Participants with a range of expertise and involvement with the EIP programme were recruited as a key component of the process evaluation, which is in line with Moore et al. [19]. Purposive sampling uses a non-representative subset of a larger population, and is constructed to serve a specific need or objective [21]. The sampling procedure was targeted specifically at key stakeholders involved in the demonstration EIP programme in three demonstration sites. A total of 40 participants were interviewed which included 22 EIP clinical team members, nine management and administrative representatives, eight service users, and one family member of a service user. Interviews were conducted in two phases, at the beginning of implementation and at a later stage of the implementation (after approximately one year). Collecting data at multiple time points can be useful as problems can be experienced during the initial the roll out of programmes which are resolved as the evaluation progresses [19]. To capture changes over time, eight participants were interviewed on two occasions at different time points.

### Procedure

HSE clinical staff and management were invited to participate through a personally addressed email sent from the research team. Keyworkers in the three demonstration sites invited service users who were known to them, and who were at an appropriate stage in their recovery to participate in this research. A semi-structured interview schedule was used to guide the interviews. This method of inquiry is valuable as it allows the preparation of questions beforehand to help direct the conversation and keep respondents on topic, and can be supplemented by follow-up questions, probes and comments. Interview questions were derived by reviewing the literature (see Supplementary file A for sample interview schedule for clinical team members). All interviews were conducted between February 2020 and January 2022. Due to the COVID-19 pandemic, interviews conducted post March 2020 were conducted virtually. Only the interviewer(s) (GN or NOC or CD; all of whom are experienced qualitative researchers post-PhD) and interviewee were present at each interview. The average interview duration was 30 min. All interviews were audio recorded and transcribed verbatim by a professional transcriber, who signed a confidentiality agreement. Field notes were written after each interview concluded. Each participant provided informed written consent prior to commencement of the interview. Confidentiality was of upmost importance in the study, as process evaluations typically involve the collection of rich data from a limited pool of participants, therefore confidentiality was assured and all data were pseudonymised and any identifiable information was removed. Participants are referred to as their main stakeholder perspective grouping and also by demonstration site (e.g. Site B, Service User). Sites A-C represent the three clinical demonstration sites, while Site D refers to administration or management which are centralised, and not located with the clinical teams. The Consolidated Criteria for Reporting Qualitative Research (COREQ) was used to improve the reporting of these findings [22] (see Supplementary file B).

### Data analysis

Content thematic analysis was utilised to identify, analyse, and report patterns or themes within the data [23]. Transcripts were analysed in an iterative process during and after the data collection period to identify the main concepts and themes. Transcripts were uploaded to the software programme NVivo (version 12) to facilitate data management, coding and retrieval. The six-phase process of coding comprised: familiarisation with the data, the generation of initial codes, searching for themes among the codes, reviewing the themes, defining and naming the themes, and production of final analysis [23]. The members of the research team (CD, NO’C, GN & HR) read the transcripts independently. Line-by-line coding was then undertaken by the researchers to assign the initial a-priori themes and relevant excerpts, and with a focus on experiential claims and concerns [24]. Patterns in the data were categorised into a thematic structure to identify and name major themes and subthemes. After the initial codes were identified and applied to the dataset, the researchers met and discussed any doubts or disagreements until consensus was reached. Data saturation was reached where no new themes emerged. Direct quotations were used to describe the themes, thereby enhancing credibility of the analysis. Transcripts were not returned to participants. For the purpose of this analysis, the focus was placed on the two most pertinent facilitators and barriers to EIP implementation reported by study participants during their interview. Further detail regarding other themes explored is available in the final project report [15].

## Results

Results coalesced around the macro level themes of facilitative factors (e.g., improvements to operational level service delivery factors and acceptance of MoC by staff and service users) and barriers (e.g., staffing and funding challenges).

### Facilitators – speed of referral, assessment and multidisciplinary approach

There was a strong sense that service users were positive about the new EIP service. A key component of this included the processes which facilitated quick referral, assessment and commencement of treatment to the multidisciplinary team. These processes continued throughout the COVID-19 pandemic using remote formats to allow for continuity of care while also complying with national public health directives. It is worth noting here that the positivity expressed by service users may be reflective of generic service change, rather than the specifics of the new EIP service. It is likely for instance that any change in a service (with its associated increased funding and staff enthusiasm) will see improvements in service user satisfaction, regardless of the specifics of the new programme. However, the interviews were structured to elicit feedback on the EIP services specifically rather than other services.

*“I got seen really quickly actually. It surprised me. I went to my GP [General Practitioner] and within the following week I had a phone call with [keyworker]. She was great. She explained everything to me. It was during COVID so she did all of the questionnaires on the phone with me. I came in later to meet with her and Dr [consultant psychiatrist]. After that I met with [cognitive behavioural therapist for psychosis (CBTp)]. That was a game changer. I am not exaggerating. They really saved my life. Without them I would not be here talking to you today. I felt so low. I also got some help with talking to my family. [Behavioural family therapist (BFT) practitioner] was amazing meeting with my Mam, I never would have been able to explain what was happening to me, how it feels to have psychosis, if I hadn’t had her [BFT practitioner] help to do that. But it all started with being seen so quickly. You hear about all those long waiting lists for people to get help, to get seen. Not for me, that was not my experience”* (Site A, Service User).

This ability to quickly refer within the EIP team structure was noted with a degree of caution by some clinicians citing the possibility of over-burdening service users with too many diverse treatments in the early stages of their care. However, it was noted that having access to a range of treatment options is important and that the EIP teams can stagger the interventions to not to overwhelm a service user while still offering an individualised approach to care.

*“I feel like we can offer them [patients] access to services now. Like a lot of the psychological services weren’t here before. Now they are here. We can get them assessed and get them set up with a treatment plan, plus make referrals within the team to who they need to see. All of that happens so much quicker now. Its great. I do think that its important to take it on a case by case basis. Some people may be ready and able to see the whole team, while others may need to have those sessions staggered”* (Site B, Clinical Lead).

Generally, clinical staff were very satisfied with the new EIP service and they were confident that they were providing a better service for people experiencing FEP than was on offer before the implementation of the service under TAU, and this was reflected in the perspectives of service users, who were positive about the services offered by the programme, as well as their experiences of engagement with these services. New roles specific to EIP such as a keyworker (or care coordinator) who coordinates all aspects of the patient’s journey through the service, and other roles such as CBTp and BFT were noted.

*“I feel like we are giving people what they need now. It’s a full wrap around service now. They are getting access to the things that they are supposed to be getting. They get to see the doctor, they get the medication, that’s reviewed. CBTp is happening and you can see the difference in them due to that. BFT [Behavioural Family Therapy] is happening and there are so many benefits to that, including the family in the treatment, including them in the ‘bigger picture’ is really key*. My role [keyworker] is new too” (Site C, Keyworker).

This was also recognised by a family member of a service user, who appreciated the multidisciplinary nature of the service and the efforts to include family as a part of the recovery process:

### Facilitative factors - enthusiasm for the principles of EIP

There was a real sense of buy-in and belief in the philosophy of EIP amongst staff and service users. The findings from across participant groups (e.g., service users, clinical team members and leadership) demonstrated that the recovery philosophy instilled in staff through the well-defined model of care works to benefit service users, who in turn developed therapeutic hope, belief in recovery and enthusiasm for the model of care. This was acknowledged by both regional and central management, highlighting how such beliefs can permeate all levels of a team, not just those with service user-facing roles. Participants routinely stated their belief in its value and worth. Both staff and service users identified several aspects of the new way of working that they believed were most effective and worthwhile. Most commonly, this was the newly devised keyworking component of the new EIP services.

*“Having one person for me to link in with. [Keyworker] is great. I talk to her every two weeks. She rings and checks in with me. We have even gone for walks together. I am not sure if that’s due to COVID or if it’s a part of it all. She organises everything and links me in with everyone else on the team. Its been really useful, especially in the early days when I was feeling really unwell and suicidal”* (Site B, Service User).

Staff described how this way of working allowed service users to receive a continuity of care that had not been previously available, allowed families and patients to have one point of contact through which a strong relationship could be built, and allowed queries and concerns to be directed to one person where they could expect a swift response. A participant welcomed the expansion of services to assess and treat the physical health of service users.

*“We are shifting, we have medical staff on board now to help with the physical assessments….that’s something that has kind of evolved. I think that is only going to put us in a stronger [position], I think that’s really good for our service as a whole and just kind of developing us going forward and I just think it shows that the need is there”* (Site D, Management).

There was a palpable sense of excitement and enthusiasm for the new MoC, the foundation of which is based upon recovery, which was seen as a refreshing perspective within the mental health service. The importance of someone championing the philosophy behind the MoC was also noted as facilitative to build and maintain momentum.

*“EIP is all about hope. It’s all about the expectation that the patient can and will get better. Its all about the importance of family in the persons recovery too. Its more than just survival and existence. It’s about the person going back to education, returning to work, returning to having their life. [Clinical Lead] was such a great champion of EIP, [Clinical Lead] would talk about the benefits of it at every meeting and what the research evidence was saying about all of the benefits that it could bring. It kept us all focusing on the ‘bigger picture’ of EIP because of [Clinical Lead] positivity about it”* (Site C, BFT).

### Barriers - obstacles to recruitment

While staff expressed enthusiasm for the concept of EIP and had noted areas where implementation had been successful, areas emerged that reflected barriers to implementation. It is worth noting that while similar facilitative themes emerged from both service users and staff at all levels, the thematic barriers emerged in relation to managerial and service process issues and were not reflected in service users’ voice and accounts.

The ability to successfully recruit and appoint clinical staff proved to be a serious challenge. In early 2019 the HSE made a decision to manage national health spending by freezing recruitment. Mental health development funding was required to meet unfunded cost growth in mental health services. As a result, nationally planned developments of EIP services had to be temporarily paused. Some of the paused funding was used for agency staffing (both medical and nursing) and to facilitate specialist placements for those with complex mental health needs. The funding became available again in 2021 for the recruitment of specialist teams. While this was not unique to EIP, it did mean that some of the enthusiasm for working within EIP eroded as doubts grew as to whether critical posts would be funded.

*“I was in the dark as to what was happening. I interviewed back in July (7 months previously) and it all went well. I was told that I had gotten the job but weeks and weeks went by and there was no further information coming. I ended up contacting [the Clinical Lead] and we had a good chat about it. They were trying to unblock it and their end but the money for the posts hadn’t come through”* (Keyworker, Site C).

Clinical leads of services described how they had been allowed to advertise positions and interview candidates, but not offer jobs as the funding was diverted temporarily to satisfy other operational needs within the health service, a practice referred to as ‘time-related savings’ which involves the retention of unspent monies in order to ensure an underspend in an area’s annual budget:

*“What happens is that we have a financial budget that we have to live with and you know constraints. For example, if I have to replace posts on 24/7 units and keep the rosters running because we have acute patients, I have an over run and it’s unfunded…there might be a decision made that I have to rely on some time related savings to be able to get me into the following year and then fill those posts in the following year….its been very difficult for people. They interviewed for these jobs, they didn’t get into the jobs for months and months, and the stuff that’s happening in the other demonstration sites is even worse, they interviewed a year ago. So how do you commit, maintain momentum, how do you get people to say ‘this is really worth investing in’. The posts are only for two years with the possible extension of three, they’re not even permanent posts. They’re not promotional, they’re all grade for grade, so all you’ve got really to offer people is you’re going to enjoy this, it’s going to be good, you’re going to develop something, it’s going to be worthwhile but it does take quite an investment of energy to keep that I think going”.*

(Site B, Clinical Lead)

In addition, there were issues around appointing staff as many of these staff members came from other parts of the health service. If a staff member applied for an EIP post, they could not take up the role until a replacement or ‘backfill’ was secured before the person could be released to start in a new EIP post. This happened particularly with keyworker roles, and led to long delays in appointments.

*“One of the most frustrating parts to this process was the backfill not being in place. When we had the money for the posts, we interviewed, we identified people to take the posts, they wanted to work here, we wanted them to work here, but they could not start as there was no backfill secured for the roles that they were leaving. This happened with two of our nurses”* (Site C, Clinical Lead).

Complications and delays surrounding the release of secured funds also played a role which is explored in the next theme.

### Barriers - budgets and permission to draw down

The ability to access the appropriate funding to appoint staff was a theme that emerged at each site and served as a major barrier to implementation. Staff had applied for agreed EIP demonstration site funding and been successful in their bid. However, a decision was taken at national level to divert these funds elsewhere. Clinical leads described long and arduous attempts at uncovering how and why monies were not made available. This financial investigation often took time and an emotional toll on clinical staff who struggled to navigate what was described as an archaic system with few forthcoming answers.

*“I couldn’t understand what was happening with these posts that we had. Whenever we were selected as one of the three demonstration sites and I couldn’t understand how is it that we can’t have these posts advertised and interviewed and what’s going on here? My understanding initially was that the money was coming through the national office, I couldn’t work out, was it that the money wasn’t given, was it that the CHO weren’t prioritising them, em was the money there, was it being used for something else? I wasted so much time and energy on trying to figure it out”.* (Site A, Clinical Lead)

Financial issues were worsened by the development of COVID-19 at the beginning of 2020, as money that had been originally ring-fenced for EIP service implementation no longer seemed to be available as the EIP service was no longer a priority. There was a sense that the national strategic programmes were being sacrificed in order to make short-term financial savings due to pressure from senior HSE management. It was also noted that mental health in particular struggles to operate services on the budgets they are allocated, partly because they receive considerably less funding than can meet demand. There was a sense that since these were long established practices within the HSE, teams, and even local and regional management, were powerless to change.

*“I guess the easy wins would be for us to within [XXXXXX] to have any additional new fundings that come in that we own that budget and that cost centre because in a central pot for operations we’re all in the same bank account. Operations will rob it because they need to do x, y and z. Whereas having a ringfenced budget would make a huge difference so we allocate the funds when they are in place to the services. We’d have much better control over it…. historically we go back to the same thing, mental health services are chronically underfunded (Site D, Management)*.

A possible solution was proposed which entailed the separation of monies relating to new development initiatives from monies associated with existing operations.

A confluence of these factors meant long delays in the release of appropriate money to each demonstration site and an unfortunate consequence of this was the draining of enthusiasm and energy in clinical leads and staff members. The timely release of promised funding was the most serious barrier to implementation of services due to the very serious implications for appropriate staffing, plus the psychological effects on enthusiastic and specialised mental health teams not being supported to deliver services efficiently.

## Discussion

The key findings of this study included the positive facilitative factors of the EIP model of rapid assessment and provision of specialist interventions alongside the belief and enthusiasm for a recovery MoC for those experiencing FEP. Balanced against this were two interrelated barriers to implementation which included staff recruitment and funding allocation, which threatened implementation of the new MoC across the three demonstration sites.

EIP services should provide the full range of treatment which is outlined in policy [25, 26]. This includes medical, psychological and social support, including support for family members. Participants recognised the importance of such an integrated approach to treatment and also how different this was compared with the traditional medical MoC that teams were in a position to offer prior to the EIP MoC being introduced at the demonstration sites. The value of the integration of services, as well as the enthusiasm for this new model of care, was reflected across participant groups (e.g., service users, clinical team members and leadership). In contrast, the primary barriers of recruitment and budgeting were only discussed by clinical teams and leadership and it is encouraging that these obstacles did not translate into major challenges for service users. Nevertheless ,the development of efficient administrative processes to facilitate the timing, sequencing and staggering of interventions may be worth considering in the full roll out of services nationwide and future service evaluations so as that the service user can benefit from those interventions during the appropriate window of their treatment trajectory. This is useful learning for new teams that care needs to be individualised and timed to the needs of service users. It is also important to note that throughout this evaluation, the facilitative factors were consistently reported by participants over follow-up while the barriers to implementation were reported more frequently over time. This may be attributed to growing frustration in the delays with recruitment and funding that became more salient at the later stages of implementation when they were long overdue.

The two main barriers to implementation identified in the current study included challenges relating to recruiting and commencing staff in posts and the ability of teams to draw down monies allocated to EIP. Workforce planning is a continuous task within many health services [27]. The constant need to create and maintain a pipeline of new graduates, recruit and retain highly trained staff and provide cover in areas of clinical and demographic need is no small feat. The challenges described in the current research did not relate to an insufficient number of trained professionals for the new roles within the three demonstration sites but rather to the permissions not being granted in a timely way to recruit staff and commence them in posts. This was exacerbated by limited contingency planning to provide backfill cover for those staff in posts that they were leaving. This is not unique to Ireland and a review found that widespread implementation of EIP services has been slow due primarily to the shortcomings regarding insufficient funding for mental health services and their low prioritisation within health systems [26]. One of the concerning knock-on effects of these barriers is that the caseloads of service providers become difficult to regulate and can exceed the limits that are designed to optimise service delivery and programme efficiency. EIP guidelines from the UK recommend a caseload of 15 patients per care coordinator and smaller caseloads are suggested as a gold standard in EIP programme delivery [28]. Although the specific caseloads were not available throughout this study, multiple service providers mentioned large caseloads and delays with recruitment as a challenge to service delivery. Similar to findings from other studies, higher caseloads led to delays in service delivery and increased burden among care teams [29]. Key working is not routine clinical practice in CMHTs in Ireland. As such, MDT members do not hold key working caseloads in CMHTs. This is distinct from practice in other jurisdictions It will be very important as EIP services grow and expand that a focus is maintained on quality assurance and fidelity to the EIP model, else EIP services risk being diluted and ineffective [30]. A focus on capacity will be crucial, and how best to utilise staff and resources effectively, so as that interventions offered to service users can be sustained and the requisite service requirements are in place to meet the demand. A follow up study of these demonstration site teams at a later more mature stage of their development would likely be valuable in identifying any persistent facilitators and barriers, or indeed any new ones.

Any new service which is implemented within an existing health service inherits the problems of that service and processes that underpin service delivery. The HSE is the largest public sector employer in Ireland, and spending on health per capita in Ireland is close to the EU average [31]. However, large system transformations can only be realised by addressing underlying problems such as financial systems, organisational prioritises, and human resource functions [13]. A radical transformation of priorities and the recognition of parity of mental health and physical health services within national public health services is required to address this. A more cohesive and organised approach to health systems funding for complex and multi-faceted initiatives is needed to integrate these services within existing local health systems structures(32). The types of resource planning for dynamic multidisciplinary teams, which coincide with CMHTs, is not as straight forward with respect to funding activity within a hospital for example. It is about getting the right number of people with the right skills in the right place at the right time to provide the right services to those that need them [33]. Existing service-level demand models tend to reflect only existing levels of service utilisation [34], which do not take into consideration fluctuating populations health needs that are not covered by current levels of service provision [35]. Recently, the WHO called on countries to consider making a paradigm shift towards the use of population health needs as the basis for health workforce planning rather than the use of current levels of health service utilisation, or simple population ratios [36]. This is likely even more important in the often complex area of mental health and especially in terms of introducing a new MoC. The teams involved within the three demonstration sites attempted to embed the new EIP MoC to allow it to find its place within the existing health service, and to operationalise activities related to recruiting staff, establishing referral pathways, ensuring and building confidence amongst staff related to the underlining therapeutic process of EIP being a recovery MoC. These activities all take time and ideally sequential planning to support implementation.

The challenges associated with a lack of appropriate funding are ongoing issues for mental health services in Ireland as well as in other jurisdictions [37]. Evaluations of mental health service provision in Ireland have reported a need to improve funding, address gaps in service provision and decrease service fragmentation [38]. The barriers associated with implementing the EIP MoC reflect many of the same issues plaguing other mental health services and highlight the need to address the resourcing and organisational aspects of service delivery [37]. Fortunately, there are examples from other jurisdictions where additional investments in mental health services produced significant improvements in key patient outcomes. Increasing providers of psychological therapy resulted in significant increases in treatment rates among those living with mental health disorders in Australia [39]. Given that EIP has been shown to be a cost-effective service and has been adopted in many jurisdictions, expansion and increased resourcing of EIP is likely to produce significant improvements in service efficiency and patient care among people living with psychosis.

Establishing efficient processes for funding and staffing will be critical to maximising the success of EIP services. As has been observed in this study and elsewhere, limitations in the availability and delivery of EIP services can create significant challenges for staff and service users and detract from the enthusiasm for this MoC [40]. One strategy that has been proposed to maximise the benefits of health system investments in mental health are clear accountability frameworks [41]. These frameworks include well-defined expectations for the cost of the service and mechanisms to ringfence funding so EIP programmes are funded to a comparable standard as other clinical programmes. Given the challenges identified in the current study regarding staffing, clear guidelines for organisational support and structures are necessary in these frameworks. This could include protected time for human resource, finance, and administrative staff to support EIP programmes, as well as clinical and health system leadership to promote the recovery-based ethos of EIP services. Also it is important to note that often clinicians have competing clinical demands. The ‘Hub and Spoke’ model operates on a synergy between the specialist EIP team and the general CMHT. There may be naturally less enthusiasm for this type of focused care on EIP from teams that have a broader portfolio of service users’ needs to meet. This may in the future undermine CMHT’s engagement with and openness to EIP. This requires ongoing monitoring and remedial steps should this occur. Support from policy makers and people within communities championing EIP services have been demonstrated to be effective approaches to developing and maintaining momentum for EIP implementation [26]. The EIP MoC is based on person-centred care and relationship-based approaches. Strong leaders who champion these principles in spite of implementation barriers has been cited as primary factor in fostering enthusiasm and optimism in EIP teams, even if they are under-resourced [40]. In the current study, EIP champions were also helpful in maintaining enthusiasm and momentum during staffing challenges. Given that the fidelity of key EIP processes (e.g., speed of referral, biopsychosocial approach) was found to be a key facilitator of EIP implementation, effective leadership, strong governance structures and the timely release of budget and pathways for recruitment of staffing, will be critical to maximising the benefits of EIP services [40] in the future roll out of EIP in Ireland. The utility of this data is immediate in the Irish context with the expected rollout of EIP services nationally, and will also be of use to teams in other jurisdictions who are considering implementing EIP for the first time or indeed expanding existing EIP services. The implementation approach in this instance were selection of three demonstrations sites. In the context of limited health resources and competing interests across the health service, the approach provides incremental value as sites and contexts from which lessons can be drawn, practice changed. The emergent information and evidence can be used to build strong business cases for more widespread implementation of EIP services across Ireland, services which are equitable, standardised and high-quality.

Strengths of this study included the evaluation of service provider and user experiences from three demonstration sites. There was strong representation from each EIP role from each demonstration site. The evaluation also commenced soon after the initiation of the service, which provided information throughout the life cycle of implementation. Unique to the context of this study, much of the data was collected during the COVID-19 pandemic and provided insights into the resilience of the service and the experiences of virtual service delivery. The results of this study may also be used as a blueprint or guidance document for the scaling-up of new EIP services throughout Ireland, as well as in other jurisdictions, although there may be specific issues at local contexts which will also need to be considered and accounted for in advance of any implementation. This study was limited in that some specific components of the MoC (e.g., individual placement and support) were implemented at the later stages or were inconsistently available to service users at some sites. This was attributed to challenges with funding and identifying eligible trained staff. This may have limited the fidelity of the MoC and detracted from the benefits of the programme for staff and service users. The barriers and facilitators described in the paper emerged at both time points and are not necessarily representative of either the beginning or the end of the study. The service users were referred to the study by staff at the demonstration sites and were not randomly recruited. As a result, they may not represent all views of those attending the service, and it is possible that there were service users who had less positive experiences. Those service users who may have had a more negative experience of the service may have been less willing to participate in study interviews and due to biases such as social desirability, may have been less likely to disclose negative experiences to the study staff [42]. In addition, service users with more severe, refractory symptoms may have struggled to participate in study interviews which may have led to an overestimation of the positive impacts of the EIP model of care. As one of the important tenets of EIP is family involvement, a limitation is that one family member of a service user was recruited to the study. COVID-19 interruptions to service provision, including redirection of funding and staff, as well as shifting to virtual delivery of some services may have attenuated some of the benefits of the MoC.

## Conclusions

Accountability frameworks that establish clear and protected processes for funding and staffing may be beneficial to mitigate organisational barriers in the health system. Ongoing data collection and programme surveillance and evaluation are also likely to improve the monitoring of program staffing needs and patient outcomes, and promote a population needs approach to EIP services. While EIP has been shown to be a cost-effective service, health systems adopting these programmes should be aware of the key facilitators and barriers to implementation in order to maximise the benefits among staff and service users experiencing FEP.

## Electronic supplementary material

Below is the link to the electronic supplementary material.

Supplementary Material 1: Sample qualitative interview schedule for EIP clinical team members

Supplementary Material 2: COREQ (Consolidated criteria for Reporting Qualitative research) Checklist

## Data Availability

The availability of qualitative data, for meta-analyses or narrative synthesis, is available on reasonable request by contacting the corresponding author.
