# Supplementary material for: Barriers and facilitators to the implementation of an early intervention in psychosis service in three demonstration sites in Ireland
Source: BMC Health Serv Res. 2023 Jun 19;23:653. doi: 10.1186/s12913-023-09585-3 (PMC10280934; doi:10.1186/s12913-023-09585-3)
Supplement: Supplementary file 1 — Supplementary Material 1: Sample qualitative interview schedule for EIP clinical team members [file 12913_2023_9585_MOESM1_ESM.docx]

**Supplementary file A: Sample qualitative interview schedule for EIP clinical team members**

Note to researcher - Before interview begins:

- Explain the nature of the research to participants (voluntary, free to stop taking part at any time, research questions, why their perceptions/opinions are important, no right/wrong answers, what the data will be used for)
- Housekeeping:
  - Recording, transcribed word for word
  - Phones on silent
  - Provide Participant Information Sheet and Consent form (e.g., confidentiality, anonymity – changing of names)
- Ask participants to sign the consent form, which will be countersigned by the researcher, retain consent forms
- Thank participants for agreeing to take part
- Start recorder

1. Can you set the context back to the beginning of the EIP service and how it all came about in the first place?
2. What problems within the current delivery of mental health services relating to the treatment of psychosis were you as a team trying to address? *(Prompts if needed: what are your aims within the service and how do these differ from the standard service, what problems are you addressing?)*
3. Can you tell me about the structure of the EIP service in relation to the ‘hub and spoke’ model? *(Prompts: what makes this different to the standard model of care?)*

1. What type of service users are you trying to target with the EIP MoC?
2. What is different in terms of your own role/work practices compared with other care programmes for psychosis that you may have worked in previously?
3. What are the most important processes in terms of delivering this MoC over others in the treatment of psychosis (*Prompts: having different team members involved like the Key Worker etc, developing shared values around holistic recovery based care)*
4. What facilitative/helpful factors do you believe has assisted in implementation of the EIP MoC so far and why? *(Prompts: to what extent are these factors generic (could be applied to other locations in the country*) or context specific (only relevant to your service in this location) (*Prompts: Enough resources, manpower, communication from the CSPD, information technology*)
5. What barriers or blocks to implementation do you believe exist and why? (*Prompts if needed: things that have hindered the setting up and running of the service in some way? Is there anything delaying or preventing implementation? E.g., manpower resources*)
6. Are there any factors that make the EIP MoC initiative particularly vulnerable to failure? If so, how?
7. In your opinion were there any unintended or unanticipated consequences – positive or negative – during the implementation phase?
8. Thinking of the EIP service as it exists now, what do you see as the three most critical strategies required to achieve successful implementation across the country in other mental health teams?
9. What would full implementation look like, and where should the focus be? (prompts if needed: In terms of the overall objective of the EIP MoC what needs to be done so that the model of care is in full operation?)
10. What is needed in your view to ‘scale up’ the EIP MoC outside of the demonstration site so they can be operational nationally?
11. Is there anything else that you would like to add?

**Thank you again for your time and willingness to participate.**
